# Extraction of Palladium(II) with a Magnetic Sorbent Based on Polyvinyl Alcohol Gel, Metallic Iron, and an Environmentally Friendly Polydentate Phosphazene-Containing Extractant

**DOI:** 10.3390/gels8080492

**Published:** 2022-08-08

**Authors:** Pavel Yudaev, Irina Butorova, Gennady Stepanov, Evgeniy Chistyakov

**Affiliations:** 1 Mendeleev University of Chemical Technology of Russia, Miusskaya Sq. 9, 125047 Moscow, Russia; 2 State Scientific Center of the Russian Federation, Institute of Chemistry and Technology of Organoelement Compounds, 111123 Moscow, Russia

**Keywords:** phosphazene, extraction, stripping, sorption, magnetic sorbent, palladium, polyvinyl alcohol, carbonyl iron, green chemistry

## Abstract

In this work, a highly efficient and environmentally friendly method for extracting palladium from hydrochloric acid media was developed. The method uses a magnetic sorbent carrying an organophosphorus extractant, which is not washed from the sorbent into the aqueous phase. The extractant was characterized by ^1^H, ^13^C, and ^31^P NMR spectroscopy and MALDI TOF mass spectrometry, and the palladium complex based on it was characterized by IR spectroscopy. According to an in vitro microbiological study, the extractant was non-toxic to soil microflora. It was established that the water uptake and saturation magnetization of the magnetic sorbent were sufficient for use in sorption processes. The sorption efficiency of palladium(II) with the developed sorbent can reach 71% in one cycle. After treatment of the spent sorbent with 5 M hydrochloric acid, palladium was completely extracted from the sorbent. The new sorbent is proposed for the extraction of palladium from hydrochloric acid media obtained by the leaching of electronic waste.

## 1. Introduction

Palladium is a noble metal of the platinum group and is widely used in various fields of science and technology. For example, palladium is used in electronics as part of multilayer ceramic capacitors of printed circuit boards [1], in the automotive industry in catalytic converters in cars [2], as well as in jewelry [3], chemical catalysis [4,5], and hydrogen energy production [6]. However, the content of palladium in natural deposits of platinum group metals is extremely low. In particular, the average palladium content in low-sulfide platinum–palladium ores from the Kievey and North Kamennik deposits is 3.32 ppm, and that in the Fedorova Tundra deposit is 1.20 ppm [7]. Therefore, the search for methods to recover palladium from industrial waste and secondary resources, such as spent automotive catalysts or waste electrical and electronic equipment (WEEE), is a promising area of research.

To date, there is no highly efficient, selective, and simple method for extracting palladium from WEEE. Pyrometallurgical processes require very high temperatures (over 1500 °C) and generate a large amount of waste and atmospheric emissions [8]. During the hydrometallurgical treatment of WEEE, a leaching solution of WEEE is prepared in concentrated hydrochloric acid in the presence of oxidizing agents, for example, aqua regia [9], which is followed by the separation and extraction of palladium(II), platinum(IV), gold(III), silver(I), copper(II), tin(II), lead(II), nickel(II), iron(II), and zinc(II) using electrodeposition, extraction, ion exchange, membrane separation, and other techniques.

Solvent extraction with organic compounds (extractants) is the most promising method for the recovery of metals from industrial waste compared to other methods due to high productivity, economic feasibility, high speed, and simple process design [10,11,12,13,14,15,16,17,18,19,20,21]. Organophosphorus extractants are becoming increasingly important in hydrometallurgical processes [22] due to their high selectivity, good solubility of both extractants and their metal complexes in nonpolar solvents, high degree of stripping, chemical stability, acid resistance, and low cost. However, a significant disadvantage of liquid organophosphorus extractants is their high toxicity. The introduction of organophosphorus extractants into a sorbent matrix would make it possible to avoid their negative impact on the environment.

To date, the processes of metal sorption by mineral (silica gel, zeolites, bentonite, activated carbon, activated alumina, and so on) and polymeric sorbents have been studied. Mineral sorbents weakly interact with metal ions and are difficult to separate from the aqueous phase and regenerate [23]. Among polymeric sorbents for metals, the most widely used are polymers containing surface hydrophilic groups capable of coordinating metals, for example, chitosan. The benefits of chitosan as a polymeric sorbent include the lack of toxicity, biocompatibility, high density of functional groups on its surface, and ease of functionalization. The drawbacks of chitosan are its low sorption capacity, sensitivity to the pH of the aqueous phase, limited reuse, poor mechanical properties, and low stability in acidic media [24]. To increase the sorption capacity, chitosan is modified with compounds containing donor nitrogen, oxygen, and sulfur atoms, which makes the process more expensive [25,26,27].

To provide for easy separation of the sorbent from the aqueous phase using a permanent magnet, magnetic particles, for example, magnetite nanoparticles, are added to the polymer along with the extractant [28,29,30]. However, the agglomeration of magnetite nanoparticles in a polymer matrix reduces the magnetic properties [31], so it is necessary to use a finely dispersed magnetic carrier.

To improve the efficiency of palladium extraction, chelate compounds containing at least two donor atoms are used. However, many chelate complexes are soluble in water, some are toxic, and due to low content of coordination sites, they poorly bind metals. Of interest are polyfunctional compounds, aryloxycyclophosphazenes, since they are biocompatible, resistant to hydrolysis in an acidic environment, and insoluble in water. The replacement of chlorine atoms in the starting chlorophosphazene produces various structures capable of metal coordination [32,33].

Here, it is proposed to use a phosphazene-containing aminophosphonate with six coordination sites as an extractant. It is planned that an extractant introduced into a magnetic gel matrix based on polyvinyl alcohol and acid-resistant carbonyl iron will effectively and selectively extract palladium(II) from hydrochloric acid media obtained by leaching WEEE and electrical capacitors.

## 2. Results and Discussion

**Figure 1:**
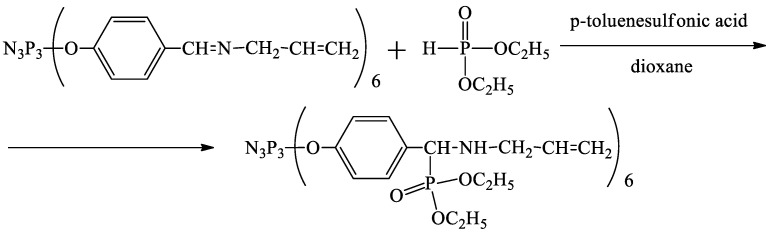
Scheme of extractant synthesis.

The extractant was synthesized by the Pudovik reaction from hexakis-[4-{(N-allylimino)methyl}-phenoxy]-cyclotriphosphazene (APP) and diethyl phosphite in dioxane, as shown in Figure 1.

The product is a light-yellow viscous mass, soluble in most organic solvents and insoluble in water, which is important in the extraction of metals from aqueous media.

**Figure 2:**
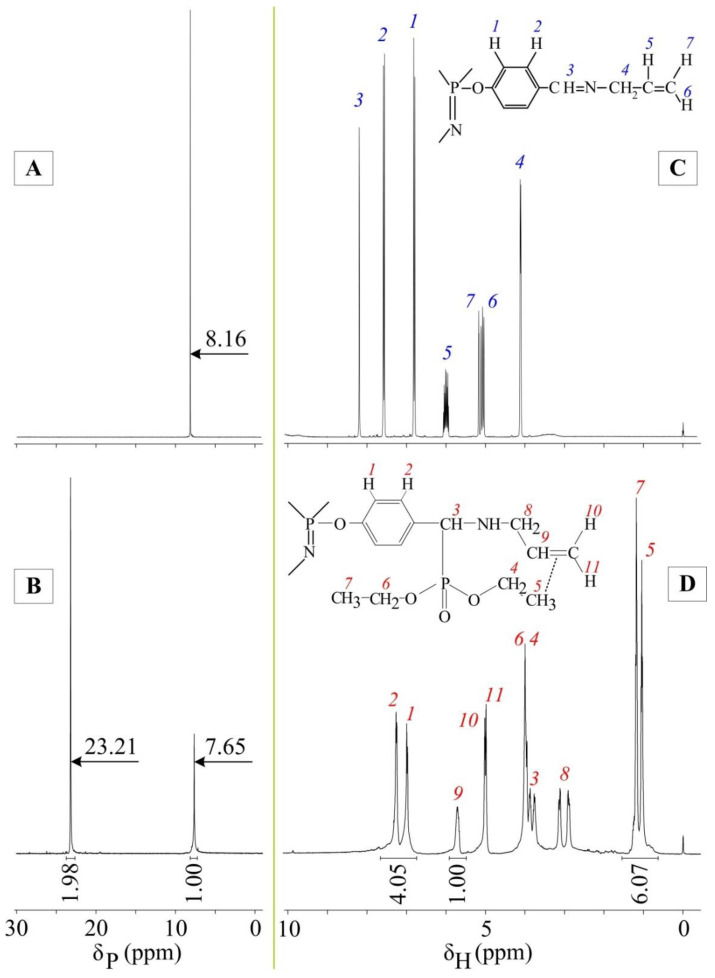
^31^P NMR spectra: APP (**A**), extractant (**B**), and ^1^H NMR spectra: APP (**C**) and extractant (**D**).

From the ^31^P NMR spectra, it can be seen that the signal of the phosphorus nuclei of the phosphazene ring of the extractant (Figure 2B) is shifted relative to the phosphorus signal of the original APP (Figure 2A) by 0.51 ppm. This is due to a decrease in the mesomeric effect acting on the phosphorus atoms due to the disruption of conjugation between the benzene rings and the azomethine nitrogen atoms, since azomethine groups have been converted to aminophosphonate groups. The formation of aminophosphonate groups is also confirmed by the presence of a phosphorus signal at 23.21 ppm. In this case, the integrated intensity ratio of the phosphorus signals of the phosphazene ring and aminophosphonate groups is approximately 1:2, which indirectly confirms the completeness of the Pudovik reaction.

**Figure 3:**
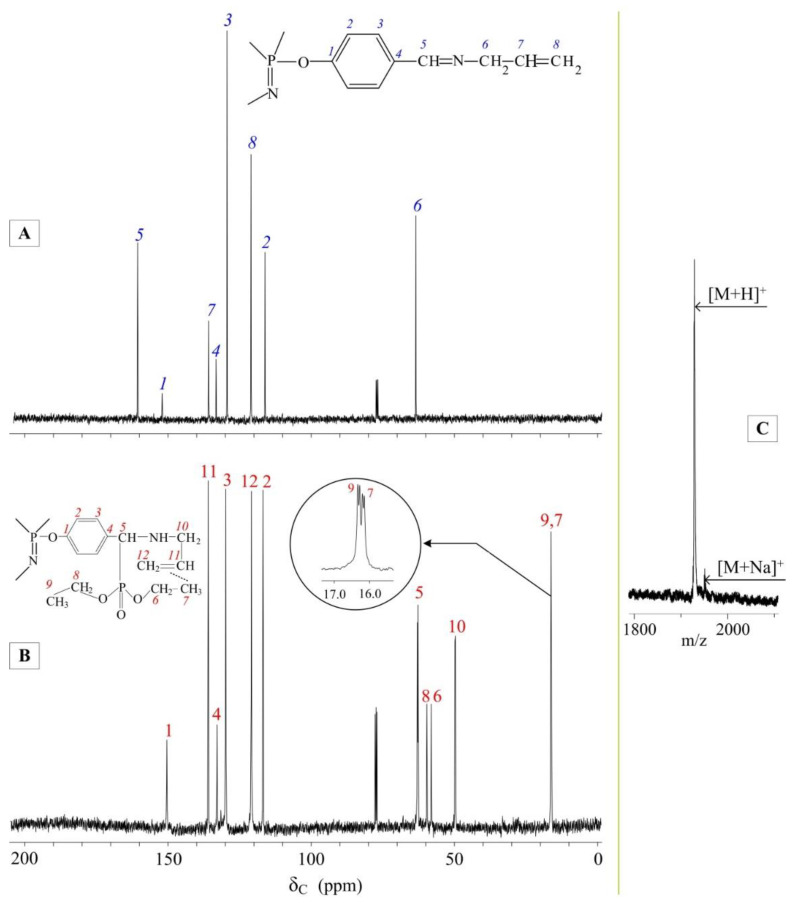
^13^C NMR spectra of APP (**A**) and extractant (**B**) and MALDI-TOF mass spectrum of extractant (**C**).

For a more accurate assessment of the conversion of azomethine groups to aminophosphonate groups, ^1^H NMR analysis was performed. It can be seen in the spectrum of the extractant (Figure 2D) that the proton signals of the azomethine groups at 8.2 ppm have completely disappeared (Figure 2C), while signals for the protons of the aminophosphonate CH groups (proton 3, Figure 2D) have appeared at 3.7–4 ppm. The integrated intensity ratio of the proton signals of the methylene groups in allyl radicals to the proton signals of the benzene ring is 1:2, which indicates the absence of side reactions involving azomethine groups during the synthesis. In addition, the number of protons of methyl groups in phosphonate radicals fully corresponds to the theoretical content, which confirms the formation of the target product. It is worth noting that the methyl proton signals form two triplets (0.98 and 1.12 ppm, Figure 2D) instead of one. The upfield shift of proton 5 (Figure 2D) relative to proton 7 is due to the contribution of the magnetic anisotropy of the double bond of allyl radicals. The signal shift of the methylene groups in the ethylphosphonate moieties in the ^1^H NMR spectrum is slight, but it is clearly visible in the carbon spectrum (carbons 6 and 8, Figure 3B). On the contrary, the difference between the carbon signals of the methyl groups is less pronounced (atoms 7 and 9). The upfield shift of the carbon 5 signal from 162 ppm (Figure 3A) to 63 ppm (Figure 3B) also indicates the complete conversion of azomethine groups to aminophosphonate groups.

The MALDI-TOF mass spectrum of the extractant (Figure 3C) shows a molecular ion peak with a solvated matrix proton in the 1925 [M + H]^+^ region, corresponding to the mass of the target compound, and a peak for sodium ion-solvated extractant at 1947 [M + Na]^+^.

**Figure 4:**
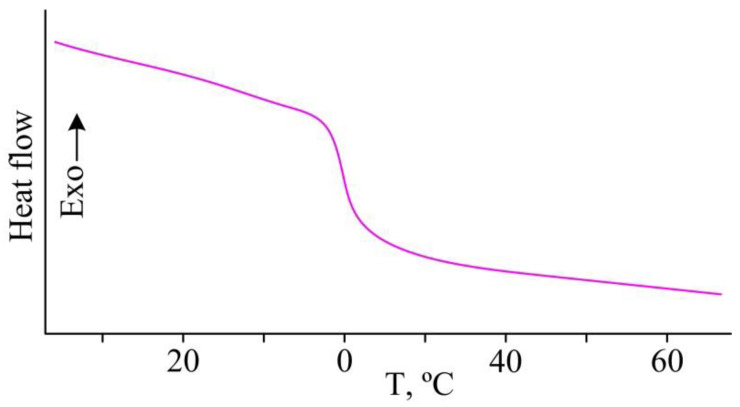
DSC curve of the extractant.

It was found by DSC that the extractant is amorphous with a glass transition temperature in the range of −5 to +5 °C (Figure 4).

**Figure 5:**
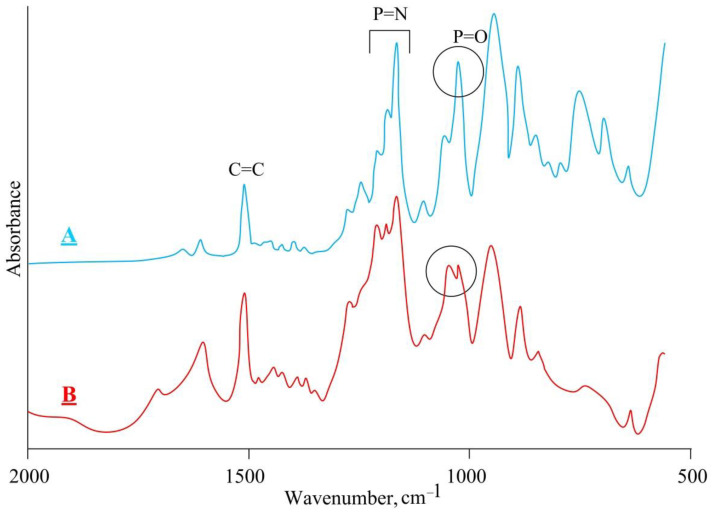
IR spectra of the extractant (**A**) and its palladium complex (**B**).

The extractant was tested in palladium extraction from chloride media. As a result of extraction, the corresponding complex was obtained, which turned out to be insoluble. A comparison of the IR spectra of the extractant (Figure 5A) and the palladium complex (Figure 5B) showed that palladium is coordinated by phosphoryl groups, as evidenced by a change in the shape of the P=O stretching band at 935 cm^−1^. It was also assumed that the double bonds of allyl groups would additionally be involved in coordination; however, the vibrational band of the double bonds of allyl groups at 1501 cm^−1^ remained unchanged.

According to the elemental analysis of the palladium complex (Table 1), there is approximately one molecule of palladium chloride per two phosphoryl groups of the extractant. This follows from the atomic ratio of phosphorus and palladium in the obtained complex, which is 3.42:1.17; i.e., it is close to the theoretical elemental ratio for the complex with the indicated structure (3.45:1.15).

**Figure 6:**
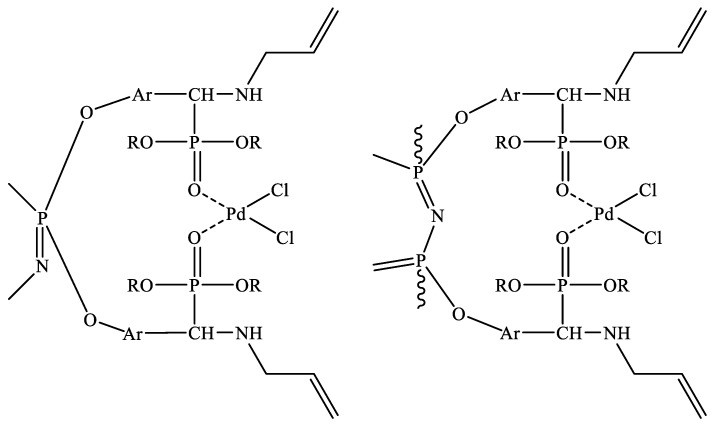
Palladium(II)-extractant chelate complexes with intra-sphere coordination of palladium (R = C_2_H_5_, Ar = *p*-C_6_H_4_).

Since palladium(II) exists in aqueous hydrochloric acid as chloride complexes PdCl_4_^2−^ [34], in the case of aminophosphonates, the extraction of palladium(II) at high acid concentrations proceeds according to the outer-sphere mechanism via protonation of the aminophosphonate nitrogen atom to give the complex {[PdCl_4_]^2−^·[HR]^+^_x_}, where R is the coordination sites. With a decrease in the concentration of hydrochloric acid, the coordination mainly follows the intra-sphere mechanism involving the chelation of palladium with phosphoryl groups. Moreover, in the case of synthesized phosphazene, palladium can be chelated by phosphoryl groups located both at the same and at different phosphorus atoms of the phosphazene ring. As a result, the formation of structurally diverse chelate complexes is possible (Figure 6).

To assess the effect of the extractant on the environment, which is important when it enters wastewater and soil, microbiological studies were carried out. It was found that when the extractant is applied to the surface of a nutrient medium inoculated with soil microflora, the extractant does not have an inhibitory effect on it. Conversely, a stimulating effect was noted compared to the control group, as evidenced by the increase in the number of microorganisms in the sample treated with the extractant (Table 2).

The stimulating effect of the extractant is probably due to the destruction of the phosphazene ring under the action of microbial enzymes and the formation of ammonium phosphates, which act as fertilizers.

Polymer sorbents were formed from a two-phase system: an extractant solution in THF and an aqueous solution of polyvinyl alcohol (PVA) with glutaraldehyde (GA). With rapid mixing of the components, the solutions gave a stable and relatively viscous emulsion, which further ensured a uniform distribution of the extractant in the polymer. The structuring of the system was provided by the addition of catalytic amounts of hydrochloric acid, while intermolecular crosslinking of polymer chains occurred due to the formation of acetals via the reaction of PVA hydroxyl groups and GA aldehyde groups.

When studying gelation, it was found that the gelation time and water absorption decrease with the increasing amount of GA added, and the amount of liquid displaced from the sorbent increases (Table 3), which is due to an increase in the degree of cross-linking of the polymer. From the obtained results, it follows that the best sorbent for use in sorption processes is sorbent number one, since it has the highest water uptake and does not displace water during gelation. Therefore, further studies were carried out using this sample.

**Figure 7:**
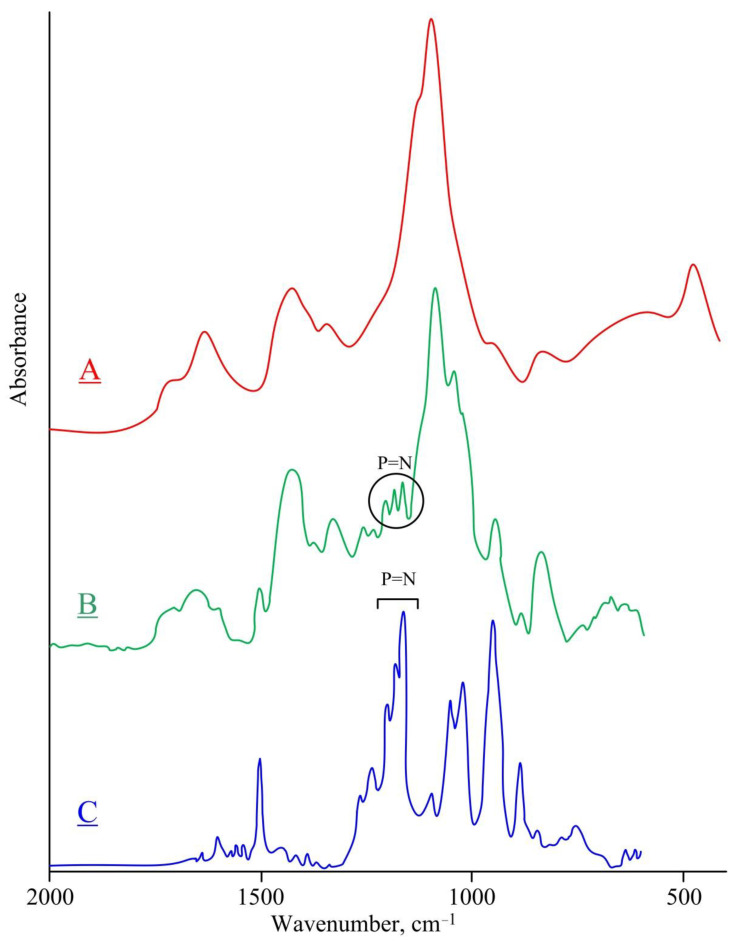
IR spectra of cross-linked PVA (**A**), sorbent (**B**), and extractant (**C**).

When conducting IR studies, it was found that the spectrum of the sorbent (Figure 7B) exhibits a vibrational band in the region of 1208–1160 cm^−1^, which is absent in the spectrum of PVA cross-linked with glutaraldehyde (Figure 7A). This band is also observed in the spectrum of the extractant (Figure 7C) and is characteristic of the stretching vibrations of the P=N units of the phosphazene ring. This fact indicates that the extractant is present in the sorbent after washing and drying, and also that the phosphazene ring has been preserved during the synthesis and isolation of the sorbent.

**Figure 8:**
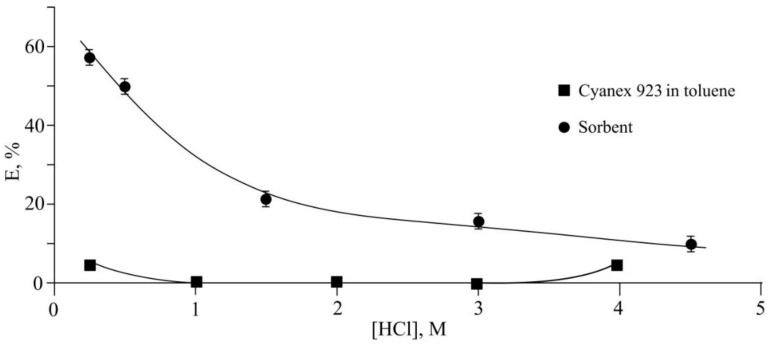
Extraction efficiency of palladium by the magnetic sorbent. In [35], palladium(II) was extracted from a hydrochloric acid medium using Cyanex 923 under the following initial conditions: [Pd] = 5 × 10^−4^ mol L^−1^, [Cyanex 923] = 0.1 mol L^−1^.

The study of the extraction properties of the sorbent showed that it is effective for the sorption of palladium(II) from aqueous hydrochloric acid solutions. It was found that the extraction efficiency increases with a decrease in the acidity of the medium and reaches 57% when a 0.25 mol L^−1^ hydrochloric acid solution is used (sorbent weight 0.1 g, volume of the aqueous phase 6 mL). This value is an order of magnitude higher than that for the liquid extraction of palladium(II) from hydrochloric acid solution with commercial monodentate extractant Cyanex 923 dissolved in toluene [35] (Figure 8). When the amount of sorbent was doubled, the extraction efficiency reached 71%.

During two cycles of extraction with one portion of the sorbent (0.1 g) for each cycle, the amount of palladium recovered reached 89%.

It was also found that 100% stripping of palladium from the sorbent is accomplished in one cycle with 5 mol L^−1^ hydrochloric acid. After stripping, the sorbent can be reused without changing the extraction efficiency.

Since production wastes and secondary raw materials containing palladium contain other metals almost in all cases, it was necessary to evaluate the extraction selectivity of the developed sorbent. For example, WEEE and electrical capacitors always contain copper together with palladium. Therefore, the Pd(II) sorption was studied from a 0.25 mol L^−1^ hydrochloric acid solution in the presence of copper(II) chloride. As a result, 52% of palladium was selectively separated by the developed sorbent in one cycle, while all copper remained in the leaching solution.

**Figure 9:**
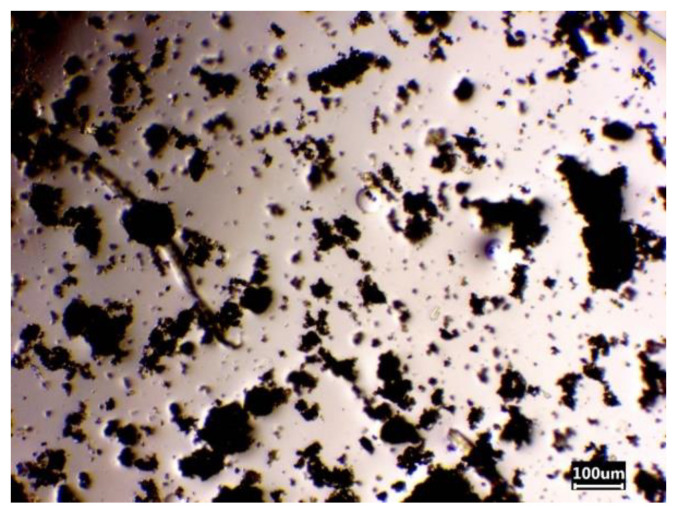
Micrograph of a magnetic sorbent film.

Magnetic properties were imparted to the sorbent by adding encapsulated iron in the gelation stage. The stability of the dispersion was ensured by the viscosity of the system. As can be seen from the micrograph of the magnetic sorbent film (Figure 9), iron particles are evenly distributed in the gel and form small agglomerates, with their linear size not exceeding 200 µm.

**Figure 10:**
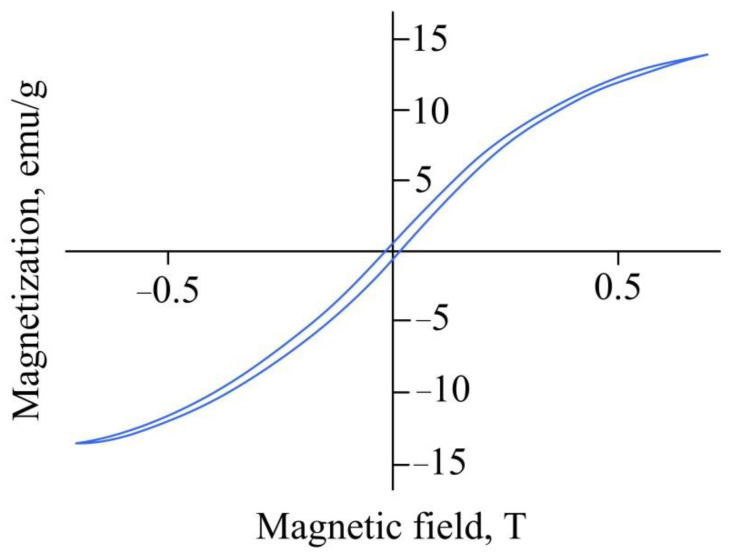
Magnetic properties of the sorbent with iron particles.

According to vibrating magnetometry data (Figure 10), the saturation magnetization of the sorbent is approximately 14 emu g^−1^. This value is sufficient for the sorbent to be separated by a magnet from water and non-magnetic particles and used in the processes of metal extraction from metallurgical waste and secondary raw materials.

The study of the properties of the magnetic sorbent showed that it has similar extraction characteristics in terms of the weight of the iron-free sorbent.

## 3. Conclusions

The new magnetic sorbent based on polyvinyl alcohol, metallic iron, and a polydentate phosphazene-containing extractant is a promising material for the solid-phase extraction of noble metals from leaching solutions of WEEE and electrical capacitors. This is due to its acid resistance, high efficiency and selectivity, excellent sorption and magnetic properties, and environmental safety. The efficiency of sorption of palladium(II) by the developed sorbent is 57% in one cycle and 89% in two sorption cycles. The spent magnetic sorbent can also be disposed of by burial in the soil, since it does not inhibit the activity of soil microflora.

## 4. Materials and Methods

### 4.1. Materials

Polyvinyl alcohol (PVA), carbonyl iron, glutaraldehyde (GA), hydrochloric acid, diethyl phosphite, *p*-toluenesulfonic acid, dioxane, tetrahydrofuran, palladium(II) chloride, copper(II) chloride, chloroform, and potassium carbonate were products of Sigma Aldrich (Saint Louis, MO, USA). Dioxane and tetrahydrofuran were dried over sodium metal followed by distillation. The encapsulation of carbonyl iron was carried out according to the procedure described in [36].

### 4.2. Methods

^1^H, ^13^C, and ^31^P NMR spectra were recorded on an Agilent/Varian Inova 400 spectrometer (Agilent Technologies, Santa Clara, CA, USA) at 400.02 MHz, 100.60 MHz, and 161.94 MHz, respectively. The mass spectrum was recorded on a Microflex LRF mass spectrometer (Bruker Daltonic GmbH, Leipzig, Germany). 3-Hydroxypicolinic acid was used as a matrix. IR spectra were measured on a Nicolet 380 spectrometer (Thermo Fisher Scientific, Waltham, MA, USA) in the spectral range of 4000–500 cm^−1^ with a wavenumber accuracy of 0.01 cm^−1^. Differential scanning calorimetry (DSC) measurements were conducted using a NETZSCH STA 449F1 instrument (Erich NETZSCH GmbH & Co. Holding KG, Selb, Germany). The hysteresis loop of a magnetic composite swollen in water was recorded using a LakeShore 7407 vibrating magnetometer (LakeShore Cryotronics Inc., Westerville, OH, USA). The distribution of iron microparticles in the polymer matrix was visually assessed using a Levenhuk MED D25T optical microscope (PRC, controlled by Levenhuk, Inc., Tampa, FL, USA). The contents of Pd(II) and Cu(II) in aqueous hydrochloric acid solutions were determined using an XSeriesII ICP-MS instrument (Thermo Fisher Scientific, USA). The composition of the palladium complex of the extractant was determined on an X-Max SDD Inca Energy Dispersive spectrometer for electron probe microanalysis (Oxford Instruments, Abingdon, UK).

Studies on the effect of the extractant on the soil microflora were carried out in vitro in flasks on a liquid nutrient medium in a shaker incubator. An enrichment culture of soil microorganisms obtained by cultivating a nutrient soil on a liquid medium of the following composition was used as an inoculation material: peptone 1.0 g L^−1^, yeast extract 0.5 g L^−1^, NaCl 0.5 g L^−1^, and glucose 2.0 g L^−1^. The medium pH was 6.5. The soil to medium ratio was 1:2. The cultivation was carried out at 30 °C for 48 h with stirring at 200 rpm. The growth of microflora was evaluated spectrophotometrically by measuring the optical density at λ = 600 nm.

To determine CFU, the method of tenfold dilutions (Koch’s method) was used. The obtained enrichment culture (1 mL) was added into flasks with 100 mL of liquid nutrient medium of the above composition, and then, the extractant diluted in 1 mL of acetone was added. Thus, the concentration of the extractant in the medium was 0.03314%. Then, 1 mL of sterile tap water was added to the control flask (control group).

After incubation at 30 °C for 48 h at 200 rpm, the resulting suspension was sown on a solid medium in Petri dishes. To do this, dilutions of the suspension were prepared in sterile tap water. An exact volume of dilution was added to Petri dishes with agarized nutrient medium and spread with a glass spatula over the surface of the nutrient medium, and colonies were counted after 1–15 days of incubation.

### 4.3. Synthesis of Hexakis-[4-{(N-allylimino)methyl}-phenoxy]-cyclotriphosphazene

Hexakis-[4-{(N-allylimino)methyl}-phenoxy]-cyclotriphosphazene (APP) was synthesized according to the procedure described in [37].

### 4.4. Synthesis of Hexakis-[4-{α,α-(N-allylamino)(O,O-diethylphosphoryl)methylidine}phenoxy]cyclotriphosphazene (Extractant)

A 50 mL round-bottom flask equipped with a reflux condenser and a magnetic stirrer was charged with APP (0.5 g, 0.4566 mmol), diethyl phosphite (0.59 mL, 0.4566 mmol), and *p*-toluenesulfonic acid (catalyst) (79 mg, 10 mol %), and the mixture was dissolved in 30 mL of dioxane. After complete dissolution, the reaction mixture was stirred at the boiling point of dioxane for 6 h in an argon atmosphere. Dioxane was distilled off, and the resulting liquid was dissolved in chloroform. Potassium carbonate (0.05 g) was added to the solution, and the mixture was stirred for 24 h at 25 °C. The solution was separated from the precipitate by decantation, and chloroform was distilled off on a rotary evaporator. The resulting substance was dried in an oven under vacuum at a temperature of 90 °C for 5 h. Yield: 0.70 g (80%).

### 4.5. Extraction of Palladium with the Developed Extractant

A solution of palladium(II) chloride (0.05 g, 0.282 mmol) in 0.5 M hydrochloric acid (3 mL) was prepared in a 10 mL glass vial. At the same time, a solution of the extractant (0.18 g, 0.0936 mmol) in chloroform (3 mL) was prepared. The extractant solution was added to the palladium(II) chloride solution and stirred at 25 °C for 48 h. The solid palladium complex formed at the interface was washed several times with distilled water and chloroform. The complex was dried under vacuum at 70 °C for 4 h.

### 4.6. Synthesis of Sorbents

Five solutions of PVA (0.8 g) in water (4.52 mL) containing 0.05, 0.1, 0.2, 0.4, and 0.8 mL of HA, respectively, were prepared in 10 mL glass vials. Five identical solutions of the extractant (0.1 g, 0.0520 mmol) in THF (1 mL) were prepared separately. Extractant solutions were added to the PVA solutions and vigorously stirred. Catalytic amounts of hydrochloric acid (3 drops) were added and stirred again. The emulsions were left at room temperature until gelation.

The gel was washed several times with distilled water and dried in a vacuum oven at 80 °C for 6 h.

### 4.7. Sorption of Palladium by the Developed Sorbent

Six solutions of palladium(II) chloride (0.012 g, 0.0677 mmol) in hydrochloric acid (6 mL) of various concentrations (0.25, 0.5, 1.5, 3, and 4.5 M) were prepared in 10 mL glass vials. Then, the sorbent (0.1 g) was placed in each vial, and the mixture was stirred for 48 h at room temperature.

### 4.8. Stripping of Palladium

The spent sorbent was treated with 5 M hydrochloric acid with stirring for 48 h at room temperature.

### 4.9. Sorption of Palladium by the Developed Gel in the Presence of Copper

A solution of palladium(II) chloride (0.012 g, 0.0677 mmol) and copper(II) chloride (0.012 g, 0.08925 mmol) in 0.25 M hydrochloric acid (6 mL) was prepared in a 10 mL glass vial. Then, the sorbent (0.1 g) was placed in the vial and stirred for 48 h at room temperature.

### 4.10. Synthesis of a Magnetic Sorbent Containing Acid-Resistant Iron

In a 10 mL glass vial, polyvinyl alcohol (0.8 g) was dissolved in distilled water (4.52 g). Then, encapsulated carbonyl iron powder (1 g) was introduced into the solution. After that, glutaraldehyde (0.05 mL), a solution of the extractant (0.1 g) in THF (1 mL), and 3 drops of hydrochloric acid were added. The mixture was stirred for about 7 min until the viscosity increased, after which it was left at room temperature until completely cured.

The gel was washed several times with distilled water and dried in a vacuum chamber at a temperature of 80 °C to constant weight.

## Figures and Tables

**Table 1:** Elemental composition of the palladium complex of the extractant, %.

Chemical Element	Actual Content		Theoretical Content	
	Weight	Atomic	Weight	Atomic
C	41.01	32.24	41.05	32.17
N	5.15	3.47	5.13	3.45
O	15.7	9.25	15.63	9.20
P	11.23	3.42	11.34	3.45
Cl	8.65	2.30	8.67	2.30
Pd	13.1	1.17	13.00	1.15
H	5.16	48.15	5.18	48.28

**Table 2:** Study of the effect of the extractant on soil microflora.

	Optical Density, Units	Number of Microorganisms, CFU mL^−1^	
		Bacteria	Yeast and Fungi
Control	9.03	8.0 × 10^9^	6.0 × 10^5^
Sample	9.29	1.29 × 10^10^	2.6 × 10^6^

**Table 3:** Parameters of synthesized sorbents.

No.	Gelation Time	Displaced Liquid, wt %	Water Uptake, wt %
1.	1 day	0	54.7
2.	1 day	23.4	53.4
3.	12 h	43.1	45.1
4.	7 h	71.6	39.4
5.	5 h	75.1	22.1
